# Is the integration of health and social care meeting the growing demand?

**DOI:** 10.12968/bjhc.2023.0081

**Published:** 2024-11-02

**Authors:** Ujhelyi Gomez, S Mechie, J Hill, K Katerina Panagaki, J Harrison

**Affiliations:** University of Liverpool; Lancashire County Council; University of Lancashire; Lancaster University; University of Lancashire

**Keywords:** delivery of healthcare, integrated care, economics, systematic review, health equity

## Abstract

Integration of care has been suggested as a solution to overcome the increasing financial and service pressures on the healthcare system. The aim of this second part of a two-part commentary is to critically evaluate a systematic review that investigated the national and international evidence base in relation to outcomes of integrated care on actual and perceived service delivery, and to identify implication for practice, policy and future research based on the quality of evidence.

## Introduction

In England, the health and social care systems operate in a legally distinct way with their own complex range of organisations, professionals, and services, and are funded accordingly (NAO, 2017). While health service provision is free at the point of access, social care packages are paid for by local authorities and are financially assessed (NHS 2022).

Today’s aging population and the increasing prevalence of complex and long-term health conditions place a great burden on the healthcare service (NHS England, 2014). Additionally, low level of social care resources due to reductions in funding (Kingston et al., 2018) has negatively impacted the healthcare system (NAO, 2017) through, for example, issues with delayed hospital discharge (Limb, 2022). Therefore, it is essential to ensure that patients receive the most cost-effective care while continuing to be patient centred. (NAO, 2017). To overcome the increasing financial and service pressures presented by these issues and the organisational, professional, legal, and regulatory boundaries within the health and social care sector, a transformation was needed in terms of the way health and social care were delivered (Crocker *et al*, 2020).

The transformation of care delivery should manifest as integrated care that is person-centred, coordinated and tailored to individual needs and preferences (NHS England, 2014). This more holistic approach involves joining up services in health and social care to work in a more collaborative way (Kelly et al., 2020). Increased service integration has also been envisaged to help achieve a health and social care system that is financially sustainable, focused on prevention and public health, empowers patients, and breaks down barriers of care (Ham and Murray, 2015). Targeting inequalities is a legal requirement in the United Kingdom (UK) and, although working collaboratively and intersectionally is the overall aim of the integrated care partnerships (Equality Act, 2010), it remains unclear and dependent on individual interpretation how this should be realised. There are a wide range of models which can be used to design and organise integrated care (Struckmann et al. 2018). Strategies to support the success of new care models include system-wide management, increased out-of-hospital care, provision of coordinated care in line with patient needs, rapid learning from good examples both nationally and internationally, and continuous evaluation of new care models (NHS England, 2014).

In its Five Year Forward View (NHS England, 2014), the NHS set out a plan to achieve the integration of health and social care services in England by 2020. Although Integrated Care Systems have existed informally since 2016 (Dunn et al. 2022), progress has been slow (NAO, 2017) and not as cost-effective as anticipated (Ahmed et al. 2015). Implementing change and assessing the new models has proved challenging and has led to uncertainty regarding the effectiveness of new care models (Castelli *et al*, 2022).

As a response, a systematic review was undertaken by Baxter and colleagues (2018) to examine the international literature regarding outcomes of integrated care on actual and perceived service delivery. To inform learning, a comparison of UK and international literature also aimed to explore similarities and differences in effects. This paper is the second of a two-part commentary, exploring solutions for the increased demands on health and social care. In a previous commentary (part 1), we critically evaluated a systematic review by Spiers et al (2019) on the relationship between social care resources and health service demands, in which the authors offered evidence to help decision-making regarding adequate funding allocation to social care. The commentary concluded that while the increase of social care supply may have the potential to ease the pressures on the healthcare system, evidence to support this is lacking (Mechie et al, 2023). An alternative solution to meet the rising demands is a more effective integration of health and social care services.

### Aims of commentary

Part 2 of this commentary aims to critically evaluate the systematic review by Baxter et al (2018) that investigated the national and international evidence base in relation to outcomes of integrated care on actual and perceived service delivery, and to contextualise the findings in regard to practice, policy, and future research.

## Methods

This protocol registered review conducted a literature search of multiple databases (including the grey literature). The reference lists of included papers were screened for relevant studies. The date range was restricted to literature published from 2006 onwards. A previous systematic review was used to identify any studies prior to 2006. An update of the search was completed in May 2017. Papers were limited to those in English or with an English abstract.

Models of integrated care were defined ‘*as changes to health or both health and health-related service delivery which aim to increase integration and/or coordination*’. Studies were included if the outcome related to the delivery of services (efficiency, effectiveness or quality of care) and/or the effect on patients and staff delivering services. Studies of any design including those with or without comparators and systematic reviews were included. Studies conducted in the UK and any other developed countries were eligible for inclusion. Studies were excluded if integrated services did not include healthcare, or service delivery outcomes were not reported.

Five percent of papers were screened by three reviewers independently to establish agreement, and the remaining 95% were screened by one reviewer with a sub-sample of 10% checked by other reviewers. Full texts of papers were then read in full and data was extracted by three reviewers. The data extractions were checked by a different team member. Due to the range of study design, a variety of tools from the six Cochrane criteria and National Institutes of Health Checklists were used to assess quality. Narrative indications of quality rather than scores were provided.

Due to the high levels of heterogeneity in terms of outcome, intervention, and design, data could not be pooled in a meta-analysis. The authors refer instead to a ‘strength of evidence approach’ to rate evidence as ‘stronger’, ‘weaker’, ‘inconsistent’, or ‘limited’. Table 1 includes the authors’ definitions of these terms. Appraisal of strength of evidence was undertaken by the research team in a series of meetings, and an overall rating of evidence was applied across all studies which reported the same outcome. Evidence was also rated separately for UK studies, systematic reviews, and international comparator and non-comparator studies, providing an overall rating of effect across the study types.

### Findings

A total of 268 articles were identified, of which 101 reported on qualitative studies and have been described elsewhere (Baxter et al, 2018). This review included the remaining 167 articles, reporting on 153 unique studies evaluating new models of integrated care. Of the included articles, 54 reported on studies conducted in the UK, 70 were international studies, and 43 were systematic reviews. Sixteen of the 54 UK studies utilised a higher quality comparator design but overall the UK studies were considered to be at risk of potential bias. While 49 of the international studies were judged to be of ‘higher quality’ due to their comparator design, none of the UK or international studies achieved all six criteria for reducing potential sources of bias.

Due to the lack of general poor quality of available studies, the authors used comparator terms to evaluate the findings. They rated the evidence as “stronger” in terms of integrated care leading to increased patient satisfaction, increased perceived quality of care, and increased/improved patient access. UK studies found a reduction in waiting times and outpatient appointments, but international evidence was found to be inconsistent for these outcomes. Evidence in relation to all other outcomes was deemed “weaker”, “inconsistent” or “very limited”.

Exploration of sub-group differences identified two of the largest patient sub-groups, older adults and those with ‘complex needs’. The strength of the findings regarding older adults were similar to those of other populations in terms of the effects of integrated care compared to the findings of the other included studies. However, in the case of patients with ‘complex needs’, “stronger evidence” of positive outcomes was found for the reduction of admissions and emergency department use in comparison to broader evidence. The studies included in the sub-group analyses used a non-comparator design, which impacts on the strength of this evidence.

### Commentary

Using the Joanna Briggs Institute Critical Appraisal tool for systematic reviews (Aromataris et al, 2015), five out of 11 criteria were judged to be satisfactory. Rather than a specific review question, a broad aim was provided resulting in a variety of outcomes to select from, which have introduced reporting bias. The study utilised appropriate quality-assessment tools, but it was unclear which tool was used to assess which study design and who performed the appraisal. From the reporting, it was difficult to determine whether screening and data extraction were performed appropriately. Publication bias was considered but not assessed which means studies with less favourable outcomes may have been under-reported. The inability to use meta-analysis was justified and it was acknowledged that included studies could not meet the standards of robust methodology, and therefore could not provide strong evidence. Due to the low quality of evidence of included studies and the bias introduced by the above limitations, the reliability and applicability of the findings are restricted.

While there is some evidence for integrated care leading to increased patient satisfaction and perceived quality of care, as well as improved patient access, better quality research is needed to determine the effectiveness of integrated care. Sub-group analysis focused on two subgroups that were reported on by the highest number of studies (older adults and those with complex needs). However, data on other populations and conditions is scarce. Moreover, there is a lack of evidence to demonstrate whether patients and their carers noticed any changes as a result of the service integration or whether they are more knowledgeable about and involved in the services. Future research should address these gaps.

Integrated care is a model of service provision which involves a range of interventions. This complexity means that it is difficult to identify how effective these interventions are in improving health through traditional systematic review methods. It has been suggested that the exploration of how interventions work, in which context and for whom may be more appropriate, which is the goal of realist reviews (Pawson *et al*, 2005). However, to obtain robust evidence to inform practice, higher quality primary research is needed using randomised controlled trials or, at a minimum, observational retrospective design to enable evidence synthesis through high-quality systematic reviews.

Access to healthcare is an important factor in determining health (NHS, 2017). Therefore, based on the recommendation of the Cochrane Handbook around considering health equity in systematic reviews (Welch et al, 2022), it would be important to investigate: a) the characteristic of individuals who experienced an increase in access to care, b) a comparison of different population groups for access to care, and c) whether those who normally struggle to get access to care have experienced any changes as a result of an integrated-care intervention, and if so, which intervention(s) brought about the change. Understanding the impact of integrated care systems on different population groups is key to reduce unfair and avoidable differences in health and to identify gaps in interventions for disadvantaged groups to prevent ill health and reduce healthcare costs (Welch et al, 2022).

While the NHS Five-Year Forward View Plan (2014) and subsequent government publications and policy (DoH, 2022) guide the move towards integrated care as the method to best manage pressures and to improve people’s experiences, this review concludes that integrated care initiatives often lead to results that can be considered positive by some while negative by others. For example, an increase in patient contact may be beneficial for patients but it also increases the costs to the health service. Therefore, the review authors suggest that rather than using integrated care approaches across the board, it may be more beneficial if policy recommendations and practice focus on approaches that target specific patient groups, for example patients with complex needs. This is in line with recommendations around ‘proportionate universalism’ according to which health action should be proportional to need while ensuring that systems are not creating further inequalities through such actions. (Marmot, 2014). Better quality evidence is needed that explores the effects of integrated care in specific populations, such as older adults.

Questions for thoughts

1) How strong is the evidence for integrated care?
2) Who could benefit more from integrated care?
3) How could health inequalities be addressed when considering integration of care?

## Figures and Tables

**Table 1:** Definition of terms used to categorise data in the evidence synthesis.

*“stronger evidence"*	*“represented generally consistent findings in multiple studies with a comparator group design, or three or more systematic reviews"*
*“weaker evidence"*	*“represented generally consistent findings in one study with a comparator group design and several non-comparator studies, or two systematic reviews, or multiple non-comparator studies"*
*“very limited evidence"*	*“represented an outcome reported by a single study"*
*“inconsistent evidence"*	*“represented an outcome where fewer than 75% of studies agreed on the direction of effect"*

## Key Points

- The evidence showing the effectiveness of integrated care in increasing patient satisfaction, perceived quality of care, and patient access is poor quality.
- Better quality evidence from primary research is required to enable the synthesis of research evidence through robust systematic reviews that can inform practice.
- Understanding the impact of integrated care systems on different population groups is key to reduce unfair and avoidable differences in health and to identify gaps in interventions for disadvantaged groups to prevent ill health and reduce healthcare costs.
- Integrated care may be more relevant for specific subgroups, such as those with complex needs, who might benefit more from this type of care provision, rather than all of health care.
- Health action should be proportional to need while ensuring that systems are not creating further inequalities through such actions.
